# Chemotherapy-induced peripheral neuropathy: longitudinal analysis of predictors for postural control

**DOI:** 10.1038/s41598-021-81902-4

**Published:** 2021-01-27

**Authors:** Jana Müller, Charlotte Kreutz, Steffen Ringhof, Maximilian Koeppel, Nikolaus Kleindienst, Georges Sam, Andreas Schneeweiss, Joachim Wiskemann, Markus Weiler

**Affiliations:** 1 Institute of Sports and Sport Science, Heidelberg University, Im Neuenheimer Feld 700, 69120 Heidelberg, Germany; 2 German Cancer Research Center, Im Neuenheimer Feld 280, 69120 Heidelberg, Germany; 3 Working Group Exercise Oncology, Division of Medical Oncology, National Center for Tumor Diseases (NCT), Heidelberg University Hospital, Im Neuenheimer Feld 460, 69120 Heidelberg, Germany; 4 Division of Physical Activity, Prevention and Cancer, German Cancer Research Center (DKFZ), National Center for Tumor Diseases (NCT), Im Neuenheimer Feld 460, 69120 Heidelberg, Germany; 5 Faculty of Medicine, Heidelberg University, Im Neuenheimer Feld 672, 69120 Heidelberg, Germany; 6 Department of Sport and Sport Science, University of Freiburg, Schwarzwaldstr. 175, 79117 Freiburg, Germany; 7 Institute of Psychiatric and Psychosomatic Psychotherapy, Central Institute of Mental Health; Medical Faculty Mannheim, Heidelberg University, Mannheim, Germany; 8 Department of Neurology, Heidelberg University Hospital, Im Neuenheimer Feld 400, 69120 Heidelberg, Germany; 9 National Center for Tumor Diseases (NCT), Heidelberg University Hospital, German Cancer Research Center (DKFZ), Im Neuenheimer Feld 460, 69120 Heidelberg, Germany

**Keywords:** Neurology, Oncology, Risk factors, Signs and symptoms

## Abstract

Impaired postural control is often observed in response to neurotoxic chemotherapy. However, potential explanatory factors other than chemotherapy-induced peripheral neuropathy (CIPN) have not been adequately considered to date due to primarily cross-sectional study designs. Our objective was to comprehensively analyze postural control during and after neurotoxic chemotherapy, and to identify potential CIPN-independent predictors for its impairment. Postural control and CIPN symptoms (EORTC QLQ-CIPN20) were longitudinally assessed before, during and three weeks after neurotoxic chemotherapy, and in three and six months follow-up examinations (N = 54). The influence of peripheral nerve function as determined by nerve conduction studies (NCS: compound motor action potentials (CMAP) and sensory action potentials (SNAP)), physical activity, and muscle strength on the change in postural control during and after chemotherapy was analyzed by multiple linear regression adjusted for age and body mass index. Postural control, CIPN signs/symptoms, and CMAP/SNAP amplitudes significantly deteriorated during chemotherapy (*p* < .01). During follow-up, patients recovered from postural instabilities (*p* < .01), whereas CIPN signs/symptoms and pathologic NCS findings persisted compared to baseline (*p* < .001). The regression model showed that low CMAP and high SNAP amplitudes at baseline predicted impairment of postural control during but not after chemotherapy. Hence, pre-therapeutically disturbed somatosensory inputs may induce adaptive processes that have compensatory effects and allow recovery of postural control while CIPN signs/symptoms and pathologic peripheral nerve function persist. Baseline NCS findings in cancer patients who receive neurotoxic chemotherapy thus might assist in delineating individual CIPN risk profiles more precisely to which specific exercise intervention programs could be tailor-made.

## Introduction

Chemotherapy-induced peripheral neuropathy (CIPN) is a common, potentially severe and dose-limiting adverse effect of cancer treatment. The compounds most commonly associated with CIPN are taxanes, platinum derivatives, and vinca alkaloids, applied either alone or as combined therapies^1^. The clinical picture of CIPN comprises sensory symptoms including tingling, burning, pain, and numbness and, in more severe cases, additional motor symptoms such as muscle cramps, weakness, and wasting^1^. In about 30% of patients, CIPN symptoms may persist for six months and longer after completion of neurotoxic chemotherapy^2^. The underlying causes of CIPN are various pathophysiological changes in the somatosensory (afferent) and motor (efferent) peripheral nerve fibers, which may lead to difficulties in postural control, concomitant with gait instabilities^3^, and an increased risk of falls^4^, associated with further medical complications, and considerably deteriorated quality of life^1^.

Several studies investigated static postural control in cancer patients in response to neurotoxic treatments, applying quantitative center of pressure (COP) analyses using a force plate^3–10^. However, available data are heterogeneous as to the time point of COP analysis, and the investigation of risk or protective factors. During taxane-based chemotherapy, postural control gradually deteriorated with increasing chemotherapy cycles and remained impaired one to three months after completion of chemotherapy^3^. Cross-sectional studies mirror this finding by showing that postural control is impaired immediately after completion of chemotherapy compared with healthy controls^7–9^. However, the longer the treatment-free period (time between completion of chemotherapy and COP analysis), the more divergent the results became^4–6^, until about ten years after completion of chemotherapy differences became no longer detectable^10^.

The pathophysiology of CIPN strongly supports a causative relationship of CIPN with impairment of postural control, but previous correlation analyses merely demonstrated low to moderate associations between various diagnostic approaches of CIPN and COP analyses^3,5,7^. Therefore, it is plausible that postural control in cancer patients treated with neurotoxic agents is additionally affected by factors other than CIPN alone, possibly including baseline peripheral nerve function, muscle strength and/or power^11^, and physical inactivity^12^.

Since comprehensive analyses on overall predictors of postural control in the context of neurotoxic chemotherapy are lacking, we extend here recently published own data^7^ by a longitudinal analysis on postural control in patients with different cancer types during and after neurotoxic chemotherapy. Specifically, the main goals of our study were to (i) determine the extent of change of postural control along with patient-reported and neurologically objectified CIPN signs/symptoms; and (ii) identify risk and protective factors that influence postural control during and after neurotoxic chemotherapy.

## Results

**Figure 1:**
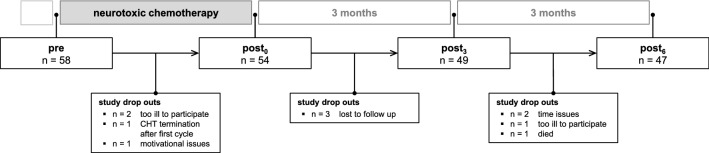
Study design and flow-chart.

A total of 58 cancer patients were included in our analysis. Four patients became ineligible after baseline testing and were excluded from further analyses (Fig. 1). Patient and treatment characteristics of the remaining 54 patients are summarized in Table 1. Thirty-seven percent of the patients were diagnosed with abnormal sensory nerve action potential (SNAP) amplitudes before starting neurotoxic chemotherapy. The proportion of patients with abnormal sensory nerve function increased with rising age: 40–49 years 16% (n = 3/19), 50–69 years 39% (n = 11/28), over 70 years 86% (n = 6/7) (Table S1). Patients were tested before (pre) and three weeks after completion of neurotoxic chemotherapy (post_0_). Between pre and post_0_ assessments, postural control and subjectively perceived CIPN symptoms were evaluated repetitively prior to each or, in case of a weekly administration schedule, prior to every second application of chemotherapy. Follow-up data were generated three (post_3_) and six months (post_6_) after post_0_ (Fig. 1). During follow-up, 26% of the patients started a structured training: sensorimotor exercise training (n = 6, mean attendance rate: 62.0%), resistance training (n = 7, mean attendance rate: 41.5%), or endurance training (n = 1, attendance rate: 100%).

**Table 1:** Patient characteristics.

	Patients
**Demographic profile**	
Number of patients [sex f:m, n]	54 (47:7)
Age [years, mean ± SD]	54.4 ± 11.6
Married [n (%)]	41 (79%)
Completed university [n (%)]	16 (31%)
**Medical profile**	
Height [cm, mean ± SD]	166.2 ± 5.9
Weight [kg, mean ± SD]	70.7 ± 13.6
BMI [kg/m^2^, mean ± SD]	25.6 ± 4.8
ECOG 0|ECOG 1 [n (%)]*	30 (56%)|12 (22%)
**Comorbidities [n (%)]**	
None	7 (13%)
Cardiovascular	19 (35%)
Musculoskeletal	32 (59%)
Neurological	4 (7%)
Endocrine/metabolic	4 (7%)
Diabetes	2 (4%)
Psychiatric	3 (6%)
**Oncological diagnosis [n (%)]**	
Breast cancer	43 (80%)
Pancreatic cancer	3 (6%)
Esophagus cancer	2 (4%)
Prostate cancer	2 (4%)
Tongue base cancer	1 (2%)
Stomach cancer	1 (2%)
Rectal cancer	1 (2%)
Ovary cancer	1 (2%)
**Disease status (UICC) [n (%)]**	
1|1A	1 (2%)|13 (24%)
2|2A|2B	1 (2%)|12 (22%)|7 (13%)
3|3A|3B|3C	0 (0%)|6 (11%)|2 (4%)|1 (2%)
4|4A|4B	6 (11%)|3 (6%)|1 (2%)
Unknown	1 (2%)
**Chemotherapy**	
Duration [weeks, mean ± SD]	17.6 ± 5.6
Time between last chemotherapy and post_0_ assessment [days, mean ± SD]	22.5 ± 9
Taxane-based [n (%)]	27 (50%)
Platinum-based [n (%)]	6 (11%)
Vinca alkaloid [n (%)]	1 (2%)
Taxane-platinum combination [n (%)]	18 (33%)
Taxane-taxane combination [n (%)]	2 (4%)
**Behavioral profile**	
Smoking [n (%)]	
Never smoker	20 (38%)
Former smoker	25 (48%)
Current smoker	7 (13%)
**Alcohol consumption (WHO) [n (%)]**	
Non-drinker (0 g/day)	18 (35%)
Harmless use (f: ≤ 12 g/day, m: ≤ 24 g/day)	28 (54%)
Harmful use (f: > 12 g/day, m: > 24 g/day)	6 (12%)

post_0_, assessment point at completion of neurotoxic chemotherapy.

*Patients with an ECOG status ≥ 2 were not included in our study. Therefore, the missing ECOG values (n = 12; 22%) are also in the range of ECOG 0–1.

### Postural control during neurotoxic chemotherapy

**Figure 2:**
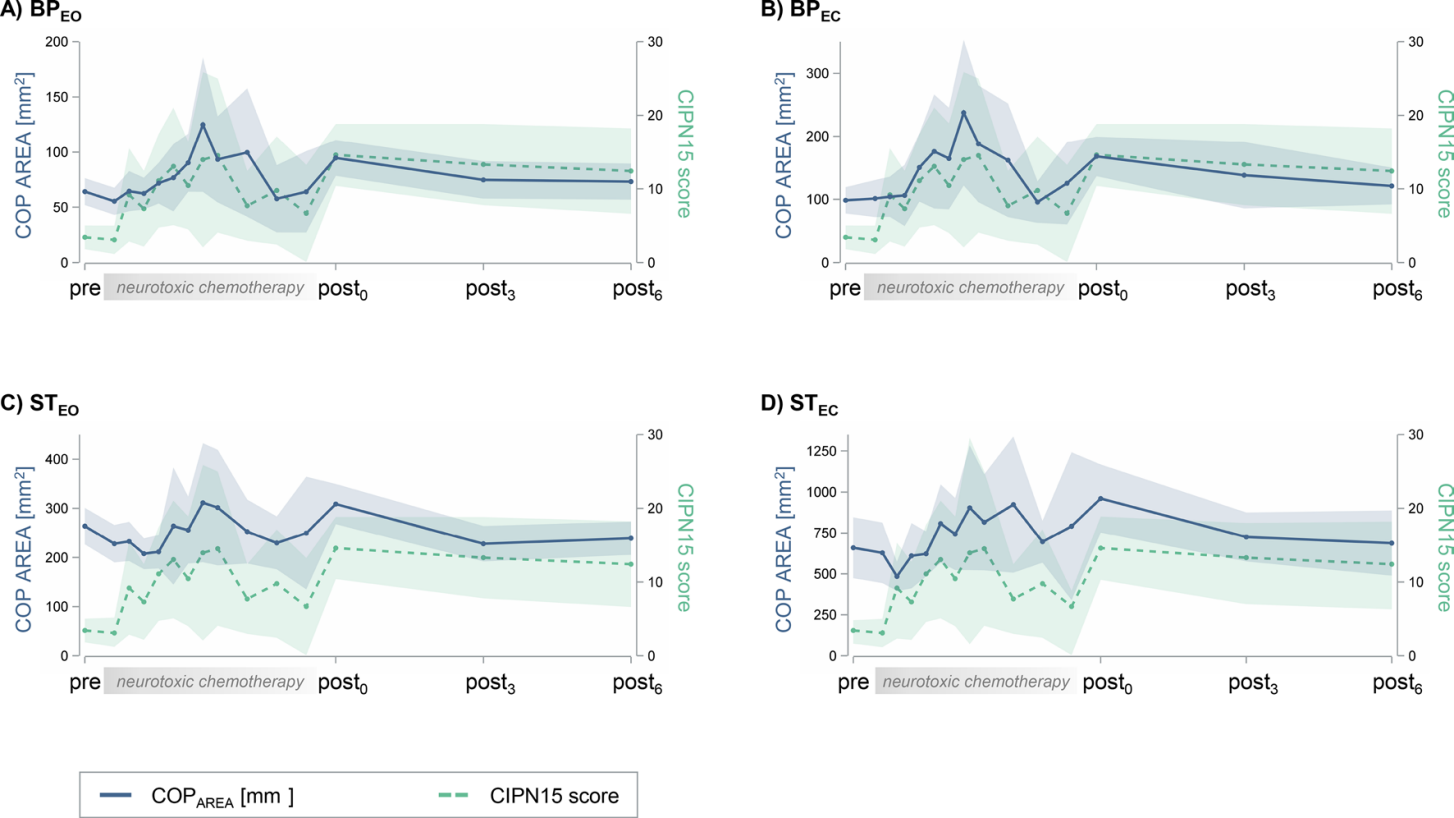
Postural control and CIPN symptoms over time. The time-scale of the data points in the ‘neurotoxic chemotherapy’ portion of the x-axis correspond to the chemotherapy cycles. Since the minimum interval between two postural control measurements was two weeks, but the individual lengths of the chemotherapy cycles in our study varied between one to three weeks, the reported sample sizes per cycle (*in brackets*) differ as follows: 32 (*2*), 34 (*3*), 34 (*4*), 32 (*5*), 18 (*6*), 21 (7*),* 7 (*8*), 14 (*9*), 13 (*11*), 10 (*13*), 7 (*15*). The blue line graphs show the averaged course (+ 95% CI, blue shading) of postural control (COP_AREA_) over the entire study period in the following standing conditions: (A) bipedal stance with eyes open (BP_EO_), (**B**) bipedal stance with eyes closed (BP_EC_), (**C**) semi-tandem stance with eyes open (ST_EO_), (**D**) semi-tandem stance with eyes closed (ST_EC_). The turquoise line graphs show the averaged EORTC-CIPN15 scores (+ 95% CI, turquoise shading) at the corresponding measurement points.

Four measurement conditions were analyzed: bipedal and semi-tandem stance, each with eyes open (BP_EO_; ST_EO_) and eyes closed (BP_EC_; ST_EC_). During neurotoxic chemotherapy, an increase in COP_AREA_ was observed with an almost parallel increase in patient-reported CIPN symptoms (Fig. 2). This descriptive observation was confirmed at the interference statistical level (Table 2): postural control deteriorated in all standing conditions (pre-post_0_: *p* < 0.0001) except for ST_EO_ (pre-post_0_: *p* = 0.04). CIPN signs/symptoms also worsened (pre-post_0_: *p* < 0.0001), as did muscle strength assessed by maximal voluntary isometric contraction (MVIC, pre-post_0_: *p* = 0.001). No significant difference was observed in physical activity behavior (PA).

**Table 2:** Descriptive statistics and results of paired t-tests.

	pre [mean ± SD]	post_0_ [mean ± SD]	post_3_ [mean ± SD]	post_6_ [mean ± SD]	pre–post_0_ [*p* value]	post_0_–post_3_ [*p* value]	post_0_–post_6_ [*p* value]	pre–post_6_ [*p* value]
**Postural control [95% confidence ellipse area]**								
BP_EO_ [mm^2^]	64.1 ± 44.2	94.7 ± 58.8	74.8 ± 51.8	73.2 ± 49.9	** < .0001** [t = 4.9; DF = 53]	.206 [t = − 1.3; DF = 38]	**.004** [t = − 3.1; DF = 38]	.345 [t = 1.0; DF = 38]
BP_EC_ [mm^2^]	98.4 ± 75.6	168.0 ± 113.6	138.3 ± 160.8	121.1 ± 88.9	** < .0001** [t = 5.4; DF = 53]	.617 [t = − 0.5; DF = 38]	** < .0001** [t = − 4.4; DF = 38]	.087 [t = 1.8; DF = 38]
ST_EO_ [mm^2^]	263.9 ± 134.7	308.7 ± 148.7	228.0 ± 110.0	239.2 ± 103.0	.042 [t = 2.1; DF = 53]	**.003** [t = − 3.2; DF = 38]	** < .0001** [t = − 4.9; DF = 38]	.097 [t = − 1.7; DF = 38]
ST_EC_ [mm^2^]	655.2 ± 673.9	943.8 ± 756.7	746.3 ± 442.9	688.6 ± 594.3	** < .0001** [t = 6.1; DF = 53]	.025 [t = − 2.2; DF = 38]	** < .0001** [t = − 5.0; DF = 38]	.862 [t = − 0.2; DF = 38]
EO_composite score_ [mm^2^]	164.0 ± 81.4	201.7 ± 91.2	151.4 ± 73.2	156.2 ± 68.3	**.002** [t = 3.2; DF = 53]	**.003** [t = − 3.2; DF = 38]	** < .0001** [t = − 5.6; DF = 38]	0.235 [t = − 1.2; DF = 38]
EC_composite score_ [mm^2^]	376.8 ± 355	555.9 ± 407.8	442.3 ± 276.1	404.9 ± 329.9	** < .0001** [t = 7.2; DF = 53]	.027 [t = − 2.2; DF = 38]	** < .0001** [t = − 6.1; DF = 38]	0.875 [t = 0.2; DF = 38]
**CIPN signs/symptoms**								
TNSc [sum score]	1.3 ± 2.1	5.8 ± 3.8	6.1 ± 4.6	5.0 ± 3.9	** < .0001** [t = 9.1; DF = 53]	.486 [t = 0.7; DF = 39]	.162 [t = − 1.4; DF = 39]	** < .0001** [t = 6.5; DF = 39]
CMAP [mV]	7.4 ± 2.9	5.5 ± 2.3	6.2 ± 2.7	5.9 ± 2.6	** < .0001** [t = − 8.0; DF = 53]	**.001** [t = 3.2; DF = 39]	** < .001** [t = 3.7; DF = 39]	** < .001** [t = − 3.8; DF = 39]
SNAP [µV]	11.3 ± 5.1	8.3 ± 5.0	9.1 ± 5.1	8.9 ± 5.5	** < .0001** [t = − 5.7; DF = 53]	.329 [t = 1.0; DF = 39]	.039 [t = 2.1; DF = 39]	** < .001** [t = − 3.4; DF = 39]
CIPN15 [sum score]	3.3 ± 5.8	14.6 ± 15.3	14.9 ± 18.4	13.3 ± 17.0	** < .0001** [t = 5.7; DF = 53]	0.793 [t = 0.3; DF = 48]	.628 [t = − 0.5; DF = 46]	** < .0001** [t = 4.4; DF = 46]
**Physical activity and strength**								
PA [min/week]	57.2 ± 94.4	35.7 ± 86.3	54.9 ± 100.6	147.7 ± 265.5	.205 [t = − 1.3; DF = 53]	.353 [t = 0.9; DF = 48]	**.002** [t = 3.3; DF = 46]	.039 [t = 2.1; DF = 46]
MVIC [Nm]	141.1 ± 34.5	131.1 ± 35.5	129.9 ± 26.0	143.2 ± 19.1	**.001** [t = − 3.2; DF = 53]	.049 [t = 2.0; DF = 27]	**.003** [t = 2.9; DF = 27]	.493 [t = − 0.7; DF = 27]

Descriptive statistics are shown for each assessment point separately (mean and standard deviation) and *p*-, t-values and DF as revealed by paired t-tests. Bold *p* values are considered statistically significant (*p* < .0125).

BP, bipedal stance; CIPN15, sum score based on EORTC QLQ-CIPN20 questionnaire; CMAP, compound muscle action potential of peroneal nerve; DF, degrees of freedom (paired t-test); EC, eyes closed; EO, eyes open; MVIC, maximal voluntary isometric contraction; PA, physical activity; pre, assessment point before neurotoxic chemotherapy; post_0_, assessment point 3 weeks after neurotoxic chemotherapy; post_3_, assessment point three months after post; post_6_, assessment point six months after post; SD, standard deviation; SNAP, sensory nerve action potential of sural nerve; ST, semi-tandem stance; t, t-value (paired t-test); TNSc, total neuropathy score (clinical).

The regression models showed that – after adjusting for age and body mass index (BMI) – high baseline SNAP amplitudes were a significant predictor for the decline in postural control in EO and EC conditions during subsequent neurotoxic chemotherapy (EO_AREA_: β = 0.37, *p* = 0.03; EC_AREA_: β = 0.44, *p* < 0.01). In EC conditions, low baseline compound muscle action potential (CMAP) amplitudes were an additional significant predictor (EC_AREA_: β = − 0.43, *p* < 0.01). All other predictors were not significant (Table 3).

**Table 3:** Multiple linear regression analysis for predicting changes in postural control during and after neurotoxic chemotherapy.

	Pre–post_0_ [n = 54]					Post_0_–post_6_ [n = 39]				
	B (95% CI)	β	t-value	*p* value	adj. R^2^	B (95% CI)	β	t-value	*p* value	adj. R^2^
**EO**					**0.12**					**0.11**
CMAP	− 3.88 (− 12.71, 4.94)	− 0.13	− 0.86	.389		− 3.47 (− 14.58, 7.64)	− 0.13	− 0.64	.529	
SNAP	6.25 (0.66, 11.84)	0.37	2.20	**.028**		− 3.85 (− 9.79, 2.09)	− 0.30	− 1.32	.196	
Age	0.8 (− 1.41, 3)	0.11	0.71	.479		− 0.01 (− 2.17, 2.16)	0.00	− 0.01	.993	
BMI	2.88 (− 2.01, 7.77)	0.16	1.15	.248		− 2.95 (− 7.56, 1.67)	− 0.24	− 1.30	.203	
PA	0.25 (− 0.01, 0.52)	0.26	1.86	.063		− 0.11 (− 0.23, 0.02)	− 0.28	− 1.77	.086	
MVIC	− 0.48 (− 1.55, 0.58)	− 0.13	− 0.89	.373		–	–	–	–	
**EC**					**0.21**					**0.11**
CMAP	− 26.33 (− 43.43, − 9.24)	− 0.43	− 3.02	**.003**		22.37 (− 7.58, 52.32)	0.31	1.52	.138	
SNAP	15.59 (4.83, 26.35)	0.44	2.84	**.005**		− 0.23 (− 16.23, 15.78)	− 0.01	− 0.03	.977	
Age	2.14 (− 2.2, 6.49)	0.14	0.97	.334		2.5 (− 3.33, 8.34)	0.15	0.87	.389	
BMI	6.36 (− 3.44, 16.15)	0.17	1.27	.204		− 7.79 (− 20.24, 4.65)	− 0.24	− 1.27	.212	
PA	0.53 (0, 1.06)	0.25	1.95	.051		− 0.05 (− 0.38, 0.28)	− 0.05	− 0.30	.769	
MVIC	− 0.61 (− 2.72, 1.51)	− 0.08	− 0.56	.575		–	–	–	–	

Results of multiple linear regression analysis investigating the influence of various predictors on changes in postural control during (pre–post_0_) and after (post_0_–post_6_) neurotoxic chemotherapy are shown. Bold *p* values are considered statistically significant (*p* < .05).

adj. R^2^, adjusted R^2^; B, unstandardized regression coefficient; β, standardized regression coefficient; BMI, body mass index; CI, 95% confidence interval; CMAP, compound muscle action potential of peroneal nerve; EC, eyes closed; EO, eyes open; MVIC, maximal voluntary isometric contraction of quadriceps [∆_pre–post0_]; PA, physical activity; post_0_, assessment point 3 weeks after neurotoxic chemotherapy; post_6_, assessment point six months after post_0_; pre, assessment point before neurotoxic chemotherapy; SNAP, sensory nerve action potential of sural nerve.

### Postural control during follow-up

Six months after completion of neurotoxic chemotherapy, patients recovered from their postural deficit (post_0_-post_6_: *p* < 0.004), thus differences to baseline assessment were no longer detectable (pre-post_6_: *p* > 0.08). Compared to post_0_, patients were more physically active (*p* < 0.01) and gained in muscle strength (*p* = 0.01). However, CIPN signs/symptoms were still worse compared to pre (pre-post_6_: *p* < 0.001; Table 2). Sub-analyses did not show different results when patients who performed a structured exercise intervention during follow-up were excluded from analyses (Table S2). The analyses of changes in postural control did not reveal any significant predictors, neither in EO nor in EC standing conditions.

## Discussion

In our study, postural control as well as patient-reported, neurologically, and electrophysiologically assessed CIPN signs/symptoms deteriorated during neurotoxic chemotherapy. Despite unchanged pathologic CIPN signs/symptoms during follow-up, postural control regenerated six months after neurotoxic chemotherapy. The regression models showed that high SNAP and low CMAP amplitudes at baseline predicted greater impairment of postural control during chemotherapy, but not during follow-up.

### Deterioration of postural control during neurotoxic chemotherapy

The deterioration of postural control concomitant with increasing CIPN signs/symptoms mirrors the results of various cross-sectional case–control studies^5,9^, randomized controlled intervention trials (e.g.^13^), and one longitudinal observational study during neurotoxic chemotherapy^3^. The latter proved deterioration in postural control in BP_EO_ and BP_EC_ within the first three cycles of a taxane-based chemotherapy^3^. In contrast, our study covered the complete chemotherapy period and included additional standing conditions. In the ST conditions, a visual improvement of postural control (lower COP_AREA_) within the first three to four chemotherapy cycles was observed. This might have been due to an initial learning effect which was not observed in the less remote BP conditions^14^. This assumed learning effect may also serve as an explanation why *p* values in the ST_EO_ condition did not survive correction for multiple comparisons. When data were corrected for this learning effect (by defining the fourth measurement as baseline), *p* values remained significant after Bonferroni correction (data not shown).

Searching for predictive factors of deterioration in postural control during neurotoxic chemotherapy, we performed a multiple linear regression analysis. Overall, the addressed predictors had a higher explanatory potential in EC than in EO conditions (adjusted R^2^ = 0.11 vs. 0.21). However, only two predictors were significant: SNAP in the EO and EC condition, and CMAP in the EC condition. Surprisingly, SNAP and CMAP amplitudes correlated inversely with the impairment of postural control: while worse baseline sensory nerve function (as expressed by low SNAP amplitudes) was a preventive factor for the impairment of postural control, worse baseline motor nerve function (as expressed by low CMAP amplitudes) predicted a greater impairment of postural control.

Vestibular, visual and especially somatosensory inputs provide the basis for a stable upright posture^15^. Hence, dysfunction of one or more of these systems may interfere with postural control but can also induce compensation processes^16^. In our study, a large proportion of patients (37%) had started neurotoxic chemotherapy with an impaired somatosensory function. This presumably age-related dysfunction^17^ might have led to postural control mechanisms such as muscular co-contraction^18^ or sensory reweighting in terms of down-weighted processing of somatosensory information and an elevated processing of visual and vestibular information to stabilize postural control (sensory reweighting theory^15,16^).

Based on these theoretical considerations, the following associations might be valid: the more the somatosensory system is impaired before chemotherapy is started (as suggested by low SNAP amplitudes), the more likely adaptive processes can be assumed—either in the sense of muscular co-contraction or reweighting of somatosensory information through central adaptation—and the less postural control gets impaired by further chemotherapy-induced damage of predominantly sensory nerves. Our follow-up data provide additional weight to this hypothesis: despite the persistence of CIPN signs/symptoms and electrophysiologically objectified peripheral nerve damage, postural control regenerated in all standing conditions six months after the end of chemotherapy.

Besides an intact somatosensory system, a sound efference (as expressed by peroneal CMAP amplitudes) is required to guarantee postural control^19^. Patients with more severe impairment of motor nerve function before starting on neurotoxic chemotherapy (e.g., due to ageing^17^) thus might be at higher risk of a deterioration in postural control during chemotherapy. Though muscle strength plays a crucial role in stabilizing an upright posture^19^, a reduction in maximum isometric quadriceps strength during neurotoxic chemotherapy in our patients does not predict a deterioration in postural control. It is likely that in our comparatively simple testing conditions the ankle strategy was primarily used to stabilize the upright posture by activating ankle plantar and dorsi flexors^20^. With increasing difficulty of the balance task, hip strategy is used and thus quadriceps and hamstrings, but especially the hip muscles, are more activated^20^. Hence, more comprehensive muscular assessments, including hip, thigh and ankle muscles, are needed to delineate more clearly the influence of muscle strength and power on postural control in CIPN patients, providing a framework for planning effective prevention measures.

Contrary to the results of exercise intervention studies in CIPN patients partly undergoing neurotoxic chemotherapy (e.g.^13^), physical activity in our patients did not show any influence on the change in postural control. However, the structured exercise programs implemented in those studies differed considerably from the self-chosen exercise efforts in our patients that were of rather low intensity, frequency, and duration. Therefore, it may be assumed that type and total load of physical activity in our patients were insufficient to influence postural control.

### Regeneration of postural deficit six months after neurotoxic chemotherapy

The observed regeneration of postural control six months after completion of chemotherapy is contrasted by unchanged pathologic CIPN signs/symptoms. Regarding previous intervention studies in CIPN patients (e.g.^21^), it is conceivable that this improvement might have been caused by enhanced physical activity during the follow-up period. However, the results of our follow-up regression analysis did not support this hypothesis. It is possible that the exercise behavior might have been too unstructured or simply ineffective in improving postural control^22^, and/or biased by recalling detailed information on this individual exercise behavior. Overall, no significant influence of the investigated predictors was found in the regression analysis.

### Practical considerations

The striking aspect of our findings is that the regeneration of postural control occurred despite persistence of CIPN signs/symptoms and electrophysiologically objectified peripheral nerve damage. Nevertheless, specific interventions for the prevention or rehabilitation of postural impairments are still indispensable. Our results do not allow a differentiation between functional regeneration and non-functional compensation, e.g. in terms of muscular co-contractions. Although co-contractions enable a safer, more stable gait in the absence of somatosensory information^23^, they also increase the risk of falling^24^. Hence, the reduction of co-contractions, for instance via a sensorimotor exercise training^25^, is desirable as a functional regeneration measure to lower the increased risk of falling in CIPN patients, associated with additional medical complications, and higher healthcare costs^4^. Moreover, in CIPN patients, a primary sensorimotor exercise training may reverse the impaired processing of somatosensory inputs by increased stimulation of less affected peripheral nerves^26^. Since SNAP and CMAP amplitudes at baseline may be predictive with regard to the extent of deterioration of postural control during neurotoxic chemotherapy, cancer patients should routinely receive thorough neurologic and electrodiagnostic examinations before starting on a neurotoxic therapy regime. These baseline findings might help to define individual CIPN risk profiles more precisely to which specific exercise intervention programs could then be tailor-made.

### Limitations and future directions

Our results are based on a sub-analysis of a larger, randomized, controlled clinical trial. Eighty-seven percent of the study participants were female, and 80% had breast cancer, so our data might be biased by sex and cancer type to some degree. However, we used comprehensive state-of-the art assessment techniques to quantify postural control as well as CIPN signs/symptoms. Moreover, we provide an unprecedented longitudinal dataset of cancer patients treated with neurotoxic agents.

Constrictively, the present sample size allowed us to analyze only a limited number of potential (partly counterintuitive) influencing factors within our regression models. Even if the ratio between predictors and analyzed subjects is sufficient, it is slightly lower than the frequently applied “one-in-ten-rule”^27,28^ deserving verification in larger sample sizes. A larger sample would also allow the omission of a composite score (dependent variable) in order to increase data interpretability and to detect potential effects on postural control strategies within different standing positions. The rather low explanation of variance yet indicates that additional influencing factors might be relevant for planning efficacious preventive and rehabilitative interventions. Moreover, future studies should extend the postural control assessment to provide further reinforcing insights into postural control strategies in CIPN patients. An important aspect is the manipulation of further sensory information, as for example shown by Monfort et al.^9^. Additionally 3D motion capture systems may be used to be able to differentiate between ankle and hip strategy in these patients^29^.

We also admit that ototoxicity which is associated with the administration of platinum-based chemotherapeutic regimens was not explicitly assessed in our study. Ototoxicity is most frequently observed with cisplatin, while carboplatin is generally less ototoxic, and ototoxicity is only very rarely seen with oxaliplatin^30^. Since the percentage of patients receiving cisplatin was very low (4%), we assume ototoxicity to play only a marginal role, if any, in our patients.

### Conclusion

The deterioration of postural control in cancer patients during neurotoxic chemotherapy may be related to baseline sensory and motor nerve functions. Six months after the completion of chemotherapy, COP parameters indicate a regeneration of postural control, while CIPN signs/symptoms persist unchanged. Whether the improvement of postural control during follow-up is based on functional regeneration or non-functional compensation strategies needs to be investigated by larger future studies.

## Methods

### Participants and study design

The cancer patients included in the present longitudinal exploratory analysis were derived from the waiting list control group of a prospective, three-armed, single-center, randomized-controlled intervention trial (PIC study; ClinicalTrials.gov identifier: NCT02871284, May 6, 2016). All experimental protocols were approved by the Ethics Committee of the Medical Faculty of the University of Heidelberg (S-630/2015, February 2, 2016). The present study represents an extension of previously published own work^7^ that compared 35 patients from the present cohort with healthy controls. The main inclusion criterion of the secondary analysis at hand was that patients received a neurotoxic chemotherapy which had not been started at the time of study assignment and baseline testing (see Table S3 for detailed inclusion and exclusion criteria). Written informed consent was obtained from all patients in accordance with The Code of Ethics of the World Medical Association (Declaration of Helsinki, 2013). During follow-up, patients were offered an exercise program within the present study (home-based sensorimotor exercise, or supervised resistance or endurance training) or to participate in another exercise intervention study (supervised endurance or resistance training; TOP-Study, ClinicalTrials.gov identifier: NCT02883699).

### Assessment procedures

All assessments were performed at the National Center for Tumor Diseases (Heidelberg, Germany). Demographic, clinical and behavioral data were collected from medical records and study-specific forms.

*Postural control* was assessed during 30 s quiet standing on a force plate (AMTI, AccuSway optimized, Watertown, USA). The detailed testing procedure is described elsewhere^7^. Four measurement conditions were analyzed: bipedal and semi-tandem stance, each with eyes open (BP_EO_, ST_EO_) and eyes closed (BP_EC_, ST_EC_). The standing positions were selected (i) to enable each patient in a (potentially) very heterogeneous population to successfully complete at least one measurement condition, (ii) to force postural control in anterior–posterior (bipedal stance) and mediolateral direction (semi-tandem stance), and (iii) to contribute to future data pooling initiatives with other studies. Additionally, the standing positions were performed with close eyes to reduce balance control to somatosensory and vestibular information. In order to avoid biased COP data due to chemotherapy-induced vertigo, acute dizziness was a test-specific exclusion criterion. The positioning of the feet in relation to each other was accurately noted for each condition in order to guarantee reproducibility in the subsequent trials. COP data were collected with a sample rate of 100 Hz and further processed in MATLAB (Version 2018a; MathWorks, Inc; Natick, MA) using custom scripts based on standard recommendations^31^. After applying a 4^th^ order Butterworth low-pass filter (cut-off: 10 Hz), 95% confidence ellipse area of the COP (COP_AREA_) was calculated to quantify balance performance. The best trial (lowest COP_AREA_ value) out of two for each condition was selected for further analyses.

*CIPN* symptoms were assessed using the patient-reported EORTC-CIPN20 questionnaire^32^. According to current recommendations, a mean sum score of 15 instead of 20 items was calculated (CIPN15: range 0–100)^33^. Additionally, CIPN signs/symptoms were assessed with the clinical version of the Total Neuropathy Score (TNSc, range 0–28)^34^. Both scores express higher CIPN signs/symptoms in higher values. In addition, nerve conduction studies (NCS) to assess CMAP of the peroneal nerve and SNAP of the sural nerve were carried out by a technician with longstanding experience in clinical neurophysiology and peripheral neuropathy. CMAP amplitudes ≤ 3.8 mV and SNAP amplitudes ≤ 9.5 µV were assessed as pathological^34^. CMAP and SNAP amplitudes are presented as average over both legs. Skin temperature was controlled at a minimum of 32 °C.

*Physical activity behavior* (PA) was assessed with a self-developed questionnaire^7^ referring to four different periods: 12 months prior to chemotherapy (pre), the time of chemotherapy (pre-post_0_) and both follow-up phases (post_0_-post_3_, post_3_-post_6_). The patients were asked to give an average of how often they exercised during these periods. Based on frequency and duration, average activity minutes per week [min/week] were calculated. Patients who participated in the training program during follow-up recorded their training sessions in training diaries. The resulting activity minutes per week were additionally added.

*Maximal voluntary isometric contraction* (MVIC) was measured for quadriceps at 36° flexion (IsoMed 2000-system B-series version, D&R Ferstl GmbH, Hemau, Germany). Patients were asked to produce maximum force over a period of six seconds. Resulting maximal peak torque was averaged over both legs.

### Statistical analysis

Statistical analyses were carried out using SAS Enterprise Guide 7.1 (SAS Institute Inc., USA). To allow for intention-to-treat analyses while avoiding bias related to imputation of data, multiple stochastic regression imputation (SAS proc MI; n = 10) was performed to impute 1.2% (pre), 2.0% (post_0_), 2.6% (post_3_) and 1.9% (post_6_) of values, which were at least missing at random. Results of the subsequent inferential statistical analyses were based on multiple imputation based on the SAS proc MIANALYZE.

Changes in postural control, CIPN signs/symptoms, MVIC and PA over study time were assessed by pairwise t-tests (1: pre-post_0_, 2: post_0_-post_3_, 3: post_0_-post_6_, 4: pre-post_6_). The level of significance was set to *p* < 0.0125 (Bonferroni-Holm corrected). The described intermediate measurements of postural control and CIPN symptoms (CIPN15 score) between pre and post_0_ were only used for descriptive illustration.

Multiple linear regression was used to analyze the relation between several predictors and the course of postural control (a) during chemotherapy (Δpre-post_0_) and (b) follow-up (Δpost_0_-post_6_). According to McCrary et al.^35^, postural control was included in the regression analyses as a composite score in order to enhance data stability^36^: time differences (Δpre-post_0_, Δpost_0_-post_6_) were calculated from the averaged COP_AREA_ of the two standing conditions with eyes open (EO_AREA_ = mean(BP_EO_, ST_EO_)) and eyes closed (EC_AREA_ = mean(BP_EC_, ST_EC_)). The following predictors were analyzed: CMAP (a: pre, b: post_0_), SNAP (a: pre, b: post_0_), PA (a: pre-post_0_, b: post_0_-post_6_), and change in muscular strength (a: Δpre-post_0_). Initially, fear of falling was also considered as a predictor, due to its associations with postural control strategies^37^. However, the individual data check revealed a distinct floor effect, and therefore this variable turned out not to be suitable as predictor variable. The analyses were adjusted for age and BMI (a: pre, b: post_0_) by including those factors in the multiple linear regression models. Supplement digital content provides additional regression analyses for each standing position separately (Table S4).

## Supplementary Information

Supplementary Information.
