# Supplementary material for: Chemotherapy-induced peripheral neuropathy: longitudinal analysis of predictors for postural control
Source: Sci Rep. 2021 Jan 27;11:2398. doi: 10.1038/s41598-021-81902-4 (PMC7840973; doi:10.1038/s41598-021-81902-4)
Supplement: Supplementary file 1 — Supplementary Information. [file 41598_2021_81902_MOESM1_ESM.pdf]

Supplementary Information

Chemotherapy-induced peripheral neuropathy: Longitudinal analysis of predictors for postural control

Supplementary Tables S1, S2, S3, and S4

Jana Müller[1,2,3], Charlotte Kreutz[4,5], Steffen Ringhof[6], Maximilian Koeppel[3], Nikolaus Kleindienst[7], Georges Sam[8], Andreas Schneeweiss[9], Joachim Wiskemann[3] & Markus Weiler[8,*]

[1]Institute of Sports and Sport Science, Heidelberg University, Im Neuenheimer Feld 700, 69120 Heidelberg, Germany

[2]German Cancer Research Center, Im Neuenheimer Feld 280, 69120 Heidelberg, Germany

[3]Working Group Exercise Oncology, Division of Medical Oncology, National Center for Tumor Diseases (NCT) and Heidelberg University Hospital, Im Neuenheimer Feld 460, 69120 Heidelberg, Germany

[4]Division of Physical Activity, Prevention and Cancer, German Cancer Research Center (DKFZ) and National Center for Tumor Diseases (NCT), Im Neuenheimer Feld 460, 69120 Heidelberg, Germany

[5]Faculty of Medicine Heidelberg, Im Neuenheimer Feld 672, 69120 Heidelberg, Germany

[6]Department of Sport and Sport Science, University of Freiburg, Schwarzwaldstr. 175, 79117 Freiburg, Germany

[7]Institute of Psychiatric and Psychosomatic Psychotherapy, Central Institute of Mental Health Mannheim, J5, 68159 Mannheim, Germany / Medical Faculty Mannheim, Heidelberg University, Germany

[8]Department of Neurology, Heidelberg University Hospital, Im Neuenheimer Feld 400, 69120 Heidelberg, Germany

[9]National Center for Tumor Diseases (NCT), Heidelberg University Hospital and German Cancer Research Center (DKFZ), Im Neuenheimer Feld 460, 69120 Heidelberg, Germany

***Corresponding author**

Markus Weiler, M.D.; phone: +49 6221 56 7504; fax: +49 6221 56 5461; email: markus.weiler@med.uni-heidelberg.de

Table S1. Sensory nerve function at baseline subdivided into three age groups.

	SNAP µV [mean ± SD]	impaired [n, %]
**< 50 years**	14.3 ± 5.0	3/19 [16%]
**50-69 years**	10.4 ± 4.4	11/28 [39%]
**≥ 70 years**	6.6 ± 2.8	6/7 [86%]

Shown are mean SNAP values at baseline (pre) subdivided into three age groups and the proportion of patients with impaired sensory nerve function according to a SNAP cut-off value of ≤ 9.5 µV.

Table S2. Descriptive statistics and results of paired t-tests, excluding patients who conducted a systematic training program during follow-up.

	**pre** [mean ± SD]	**post$_0$** [mean ± SD]	**post$_3$** [mean ± SD]	**post$_6$** [mean ± SD]	**pre - post$_0$** [p-value]	**post$_0$ - post$_3$** [p-value]	**post$_0$ - post$_6$** [p-value]	**pre - post$_6$** [p-value]
**Postural control** [95% confidence ellipse area]								
BP_{EO} [mm²]	64.1 ± 44.2	94.7 ± 58.8	76.4 ± 52.3	80 ± 54.7	**<.0001** [t = 4.9; DF = 53]	.070 [t = -1.9; DF = 35]	**.002** [t = -3.4; DF = 26]	.574 [t = 0.6; DF = 26]
BP_{EC} [mm²]	98.4 ± 75.6	168.0 ± 113.6	155.3 ± 185.7	141.1 ± 96.3	**<.0001** [t = 5.4; DF = 53]	.609 [t = -0.5; DF = 35]	**<.0001** [t = -4.1; DF = 26]	.155 [t = 1.5; DF = 26]
ST_{EO} [mm²]	263.9 ± 134.7	308.7 ± 148.7	223.4 ± 84.1	247.0 ± 102.9	.042 [t = 2.1; DF = 53]	**.001** [t = -3.8; DF = 35]	**<.0001** [t = -5.1; DF = 26]	.045 [t = -2.1; DF = 26]
ST_{EC} [mm²]	655.2 ± 673.9	943.8 ± 756.7	762.6 ± 460.3	748.4 ± 673.6	**<.0001** [t = 6.1; DF = 53]	.024 [t = -2.3; DF = 35]	**<.0001** [t = -3.8; DF = 26]	.558 [t = -0.6; DF = 26]
$EO_{composite\ score}$ [mm²]	164.0 ± 81.4	201.7 ± 91.2	149.9 ± 57.2	163.5 ± 69.9	**.002** [t = 3.2; DF = 53]	**<.0001** [t = -4.1; DF = 35]	**<.0001** [t = -5.9; DF = 26]	.099 [t = -1.7; DF = 26]
$EC_{composite\ score}$ [mm²]	376.8 ± 355	555.9 ± 407.8	459.0 ± 295.2	444.8 ± 373.6	**<.0001** [t = 7.2; DF = 53]	.025 [t = -2.2; DF = 35]	**<.0001** [t = -4.8; DF = 26]	.771 [t = -0.3; DF = 26]
**CIPN signs/symptoms**								
TNSc [sum score]	1.3 ± 2.1	5.8 ± 3.8	7.1 ± 4.8	5.9 ± 4.2	**<.0001** [t = 9.1; DF = 53]	.368 [t = 0.9; DF = 36]	.932 [t = -0.1; DF = 27]	**<.0001** [t = 6; DF = 27]
CMAP [mV]	7.4 ± 2.9	5.5 ± 2.3	6.4 ± 2.3	6.2 ± 2.4	**<.0001** [t = -8.0; DF = 53]	**.001** [t = 3.2; DF = 36]	**<.0001** [t = 4.3; DF = 27]	**.005** [t = -2.8; DF = 27]
SNAP [µV]	11.3 ± 5.1	8.3 ± 5.0	9.6 ± 5.7	8.9 ± 6.0	**<.0001** [t = -5.7; DF = 53]	.316 [t = 1; DF = 36]	.111 [t = 1.6; DF = 27]	**.005** [t = -2.8; DF = 27]
CIPN15 [sum score]	3.3 ± 5.8	14.6 ± 15.3	17.3 ± 20.4	15.3 ± 19.0	**<.0001** [t = 5.7; DF = 53]	.834 [t = 0.2; DF = 44]	.741 [t = 0.3; DF = 33]	**<.0001** [t = 3.6; DF = 33]
**Physical activity and strength**								
PA [min/week]	57.2 ± 94.4	35.7 ± 86.3	33.8 ± 55.5	115.7 ± 267.4	.205 [t = -1.3; DF = 53]	.786 [t = 0.3; DF = 44]	.014 [t = 2.6; DF = 33]	.190 [t = 1.3; DF = 33]
MVIC [Nm]	141.1 ± 34.5	131.1 ± 35.5	131.6 ± 29.2	143.3 ± 18.9	**.001** [t = -3.2; DF = 53]	.086 [t = 1.7; DF = 24]	.303 [t = 1; DF = 17]	.411 [t = -0.8; DF = 17]

Descriptive statistics are shown for each assessment point separately (mean and standard deviation) and p-values, t-values and DF as revealed by paired t-tests. Bold p-values are considered statistically significant different ($p<.0125$). **Abbreviations**: **BP**, bipedal stance; **CIPN15**, sum score based on EORTC QLQ-CIPN20 questionnaire; **CMAP**, compound muscle action potential of peroneal nerve; **DF**, degrees of freedom (paired t-test); **EC**, eyes closed; **EO**, eyes open; **MVIC**, maximal voluntary isometric contraction; **PA**, physical activity; **pre**, assessment point before neurotoxic chemotherapy; **post$_0$**, assessment point three weeks after neurotoxic chemotherapy; **post$_3$**, assessment point three months after post$_0$; **post$_6$**, assessment point six months after post; **SD**, standard deviation; **SNAP**, sensory nerve action potential of sural nerve; **ST**, semi-tandem stance; **t**, t-value (paired t-test); **TNSc**, total neuropathy score (clinical).

Table S3. Patients' inclusion and exclusion criteria.

**Inclusion criteria**	▪ age ≥ 18 years<br>▪ diagnosed with cancer and assigned to receive a chemotherapeutic regimen containing at least one of the following agents:<br>- a platinum analog, e.g. cisplatin, carboplatin, oxaliplatin<br>- a vinca alkaloid, e.g. vincristine<br>- a taxane, e.g. paclitaxel, docetaxel<br>- suramin<br>- thalidomide or lenalidomide<br>- bortezomib<br>▪ physical capability that allows the performance of the training program implemented within the experimental intervention or the control intervention arm
**Exclusion criteria**	▪ known polyneuropathy of any kind or any polyneuropathic signs or symptoms at baseline<br>▪ family history positive for any hereditary polyneuropathy<br>▪ known metastasis to the central or peripheral nervous system<br>▪ any physical or mental handicap that would hamper the performance of the training program implemented within the intervention arms<br>▪ known history of alcohol or illegal drug abuse or any constellation of lab values suggesting alcoholism, e.g. elevated GGT, MCV, CDT

Table S4. Multiple regression analysis for predicting the change in postural control during and after neurotoxic chemotherapy.

	pre - $post_0$					$post_0$ - $post_6$				
	**B (95% CI)**	**β**	**t-value**	**p-value**	**adj. R^2**	**B (95% CI)**	**β**	**t-value**	**p-value**	**adj. R^2**
**BP_{EO}**					**-0.03**					**-0.08**
CMAP	-2.17 (-7.11, 2.77)	-0.14	-0.86	.389		-4.44 (-13.49, 4.62)	-0.22	-1.00	.326	
SNAP	2.16 (-0.91, 5.24)	0.24	1.38	.168		0.15 (-4.69, 4.99)	0.02	0.06	.950	
age	0.04 (-1.21, 1.29)	0.01	0.07	.947		0.48 (-1.29, 2.24)	0.11	0.55	.585	
BMI	0.98 (-1.81, 3.76)	0.10	0.69	.492		-1.38 (-5.14, 2.39)	-0.15	-0.74	.463	
PA	0.09 (-0.07, 0.24)	0.16	1.11	.268		0.01 (-0.09, 0.11)	0.03	0.16	.870	
MVIC [$\Delta_{pre-post}$]	-0.01 (-0.62, 0.6)	0.00	-0.03	.979		-	-	-	-	
**BP_{EC}**					**0.07**					**-0.06**
CMAP	-5.29 (-15.09, 4.51)	-0.16	-1.06	.290		-8.4 (-24.65, 7.86)	-0.23	-1.05	.301	
SNAP	4.33 (-1.8, 10.46)	0.23	1.38	.166		3.44 (-5.25, 12.13)	0.20	0.81	.426	
age	1.27 (-1.21, 3.74)	0.15	1.00	.315		0.68 (-2.49, 3.84)	0.08	0.43	.667	
BMI	6.68 (1.18, 12.18)	0.34	2.38	**.017**		-3.05 (-9.8, 3.70)	-0.19	-0.92	.365	
PA	0.2 (-0.1, 0.5)	0.18	1.29	.199		-0.05 (-0.23, 0.13)	-0.10	-0.57	.570	
MVIC [$\Delta_{pre-post}$]	0.1 (-1.1, 1.3)	0.02	0.16	.871		-	-	-	-	
**ST_{EO}**					**0.08**					**0.11**
CMAP	-5.59 (-22.01, 10.83)	-0.10	-0.67	.504		-2.51 (-22.67, 17.65)	-0.05	-0.25	.802	
SNAP	10.35 (-0.02, 20.71)	0.33	1.96	.050		-7.86 (-18.63, 2.92)	-0.34	-1.48	.147	
age	1.55 (-2.57, 5.67)	0.11	0.74	.460		-0.5 (-4.42, 3.43)	-0.05	-0.26	.799	
BMI	4.78 (-4.36, 13.92)	0.15	1.03	.305		-4.52 (-12.9, 3.86)	-0.20	-1.10	.280	
PA	0.42 (-0.08, 0.92)	0.23	1.65	.099		-0.22 (-0.44, 0)	-0.32	-2.03	.051	
MVIC [$\Delta_{pre-post}$]	-0.96 (-2.96, 1.04)	-0.14	-0.94	.346		-	-	-	-	
**ST_{EC}**					**0.14**					**0.09**
CMAP	-47.37 (-81.26, -13.49)	-0.41	-2.74	**.006**		53.14 (-7.41, 113.69)	0.37	1.79	.083	
SNAP	26.85 (5.54, 48.15)	0.40	2.47	**.014**		-3.89 (-36.25, 28.47)	-0.06	-0.24	.808	
age	3.02 (-5.63, 11.67)	0.10	0.69	.493		4.33 (-7.46, 16.13)	0.13	0.75	.460	
BMI	6.03 (-13.47, 25.54)	0.09	0.61	.544		-12.54 (-37.7, 12.63)	-0.19	-1.01	.318	
PA	0.86 (-0.2, 1.91)	0.22	1.59	.111		-0.05 (-0.71, 0.62)	-0.02	-0.14	.890	
MVIC [$\Delta_{pre-post}$]	-1.31 (-5.51, 2.89)	-0.09	-0.61	.541		-	-	-	-	

Shown are results of multiple linear regression analyses investigating the influence of various predictors on changes in postural control during (pre - $post_0$) and after ($post_0$ - $post_6$) neurotoxic chemotherapy. Bold p-values are considered statistically significant different ($p<.05$). Abbreviations: **Adj. R^2**, adjusted R^2; **B**, unstandardized regression coefficient ; **β**, standardized regression coefficient; **BMI**, body mass index; **BP**, bipedal stance; **CI**, 95% confidence interval; **CMAP**, compound muscle action potential of peroneal nerve; **EC**, eyes closed; **EO**, eyes open; **MVIC**, maximal voluntary isometric contraction of quadriceps; **PA**, physical activity; **$post_0$**, assessment point three weeks after neurotoxic chemotherapy; **$post_6$**, assessment point six months after post; **pre**, assessment point before neurotoxic chemotherapy; **SNAP**, sensory nerve action potential of sural nerve; **ST**, semi-tandem stance.
